# Defining the symptoms of personality and behaviour changes in brain tumour patients and their impact on caregivers

**DOI:** 10.1007/s00520-025-09966-w

**Published:** 2025-10-17

**Authors:** Emma McDougall, Haryana M. Dhillon, Karin Piil, Lauren J. Breen, Anna K. Nowak, Sara Nordentoft, Sine Kjærgaard, Georgia K. B. Halkett

**Affiliations:** 1 Faculty of Health Sciences, Curtin School of Nursing, Curtin University, Perth, Australia; 2 Psycho-Oncology Cooperative Research Group, School of Psychology Faculty of Science, The University of Sydney, Camperdown, Australia; 3 Faculty of Health and Medical Sciences, Department of Clinical Medicine, University of Copenhagen, Copenhagen, Denmark; 4 Department of Oncology, Centre for Cancer and Organ Diseases, Copenhagen University Hospital; Rigshospitalet, Copenhagen, Denmark; 5 Curtin School of Population Health, Curtin University, Perth, Australia; 6 Curtin enAble Institute, Curtin University, Perth, Australia; 7 Medical School, University of Western Australia, Perth, WA Australia; 8 Department of Neurosurgery, Copenhagen University Hospital, Copenhagen, Denmark; 9 Curtin Medical Research Institute, Curtin University, Perth, Australia

**Keywords:** Brain tumour, Personality and behaviour changes, Qualitative research, Healthcare professionals, Carers

## Abstract

**Purpose:**

This study aimed to (i) identify how carers and healthcare professionals define and describe brain tumour–related personality and behaviour changes in adults and (ii) explore the impact of these changes on family carers.

**Methods:**

We conducted 22 semi-structured interviews with neuro-oncology healthcare professionals working in Australia and 13 interviews with current or bereaved carers in Australia and Denmark. Definition data were analysed deductively using the Diagnostic and Statistics Manual of Mental Disorders 5th Edition Text Revision (DSM-5-TR) Personality Change Due to Another Medical Condition diagnostic criteria, and inductively to uncover new ideas related to descriptions of brain tumour–related personality and behaviour changes perceived by carers and healthcare professionals. Inductive thematic analysis was conducted to develop themes exploring the impact of patient personality and behaviour changes on carers.

**Results:**

Two themes were identified from participants’ description of personality and behaviour changes associated with brain tumours: (i) variable presentation and multifactorial aetiology and (ii) manageable versus challenging changes. The specific features and symptoms of personality and behaviour changes reported by the sample are presented alongside the DSM-5-TR features of Personality Change Due to Another Medical Condition for clinical utility.

Three themes were identified related to the impact of patient brain tumour–related personality and behaviour changes on carers: (i) grieving the person: “I don’t know who this person is anymore”; (ii) bearing the brunt of behavioural changes: “Happening behind closed doors”; and (iii) safety concerns and aggression: “I didn’t know what was going to happen next”. The three main themes were further elaborated through corresponding subthemes.

**Conclusions:**

This study highlights the nuances and complexity in conceptualising personality changes in patients with a brain tumour and the grief, isolation, and safety concerns experienced by carers. Brain tumour–related aggression was identified as a significant concern by both healthcare professionals and carers, lacking clinical guidelines internationally for managing violence and aggression in this population. Future research is required to test interventions and support for safeguarding and risk management for patients and their family members.

## Introduction

Personality and behaviour changes are estimated to occur in approximately 67% of patients with glioma [1]. Effective management for brain tumour–related personality and behaviour changes (BTrPBc) is difficult due to the diverse contributing factors. Further, there is no universally accepted definition of BTrPBc [1]. In the Diagnostic and Statistical Manual of Mental Disorders (DSM-5-TR) [2], the diagnostic category Personality Change Due to Another Medical Condition is defined as “a persistent personality disturbance that represents a change from the individual’s previous characteristic personality pattern due to the direct pathophysiological effects of a medical condition”. The diagnostic features include aggression, apathy, paranoia, disinhibition, and affective lability.

Recent research led to the development of a working definition for behavioural and personality changes in adults with glioma:

“An alteration of personality and behaviour, which can be caused by a glioma and/or its treatment, and may vary in severity, frequency and magnitude during the disease process. This alteration of personality and behaviour comprises significant changes in (1) emotions, needs and impulses such as loss of emotional control, decreased motivation or initiative, and indifference, (2) changes in personality traits such as being more selfish, obsessive, or inflexible, and (3) poor judgement abilities” [3].

A systematic review on the prevalence of personality and behaviour changes in adults with glioma yielded nine qualitative studies which highlighted personality changes as distressing and challenging for carers to navigate, often impacting the relationship between patient and carer, and requiring adjustment over time [1]. One study suggested interventions should be focused on patient awareness of changes and patient and carer communication [4]. However, further research is needed to understand the perspectives of those providing care for patients (carers and healthcare professionals) to inform a more nuanced conceptualisation of BTrPBc.

Specifically, the impact on carers of supporting a patient with BTrPBc has not been explored via in-depth interviews. To address this gap, we aimed to (i) identify how healthcare professionals and carers define and describe BTrPBc in adults and (ii) explore the perceived impact of patient BTrPBc on carers.

## Methods

Ethics approval was obtained from Curtin Human Research Ethics Committee (HRE2022-0296). This study follows the Declaration of Helsinki and is registered at the Danish Data Protection Agency (Jr. nr p-2024–15544).

### Theoretical framework

This qualitative study adopted a phenomenological framework [5] to explore the descriptions of BTrPBc and impact of these changes on carers. This approach aims to uncover insights into how an individual perceives and makes meaning of a phenomenon providing rich, detailed exploration of their lived experiences. A phenomenological framework accepts the interaction between the participant’s narrative and the interpretative framework on the part of the researcher as central to the analytic process [6]. HCP interviews were conducted by EM. Carer interviews were conducted by KP, SK (neuro-oncology nurses), SN (neurosurgery nurse), and EM (clinical psychologist), who all have experience with qualitative interviewing. We have reported on carers unspoken thoughts elsewhere [7] and healthcare professionals’ management of personality changes, including factors that facilitate and encumber practice [8].

### Participants

Healthcare professionals (HCPs) and carers were included in this sample to comprehensively address the study aims and ensure a rich and nuanced understanding accounting for both clinical and lived experiences.

Eligible participants included current and bereaved carers of a patient with brain tumour–related personality and behaviour changes. Current and bereaved carers were interviewed in Australia and Denmark between May and December 2024.

HCPs who currently worked with brain tumour patients in Australia were eligible to participate. HCPs were interviewed in Australia from August to December 2023.

Identification codes were allocated to each participant to de-identify the data. “HCP” = Healthcare professional and their profession is provided, “C” represents current carers, “BC” represents bereaved carers, DK = Denmark, Aus = Australia. Demographics and clinical experience (of HCPs) are detailed in Tables 1 and 2.

**Table 1:** Healthcare professional sample characteristics (*N* = 22)

	*N*	%
**Sex**		
Male	2	9
Female	20	91
**State/region**		
Australian Capital Territory	1	5
New South Wales	6	27
Queensland	6	27
Victoria	5	23
Western Australia	4	18
**Clinical setting**		
Tertiary referral cancer centre	16	73
District/local hospital	2	9
Non-hospital practice	4	18
**Primary profession**		
Cancer care coordinator^a^	11	50
Clinical psychologist or neuropsychologist	4	18
Radiation oncologist	2	9
Neurosurgeon	1	5
Radiation oncology nurse	1	5
Occupational therapist	1	5
Psychiatrist	1	5
Speech pathologist	1	5
**FTE**^b^** in clinical practice**		
Full time-1.0	9	41
≥ 0.5	8	36
< 0.5	5	23
**Years in profession**		
2–10	2	10
11–20	10	45
> 21	10	45
**Caseload of primary brain tumour patients**		
0–25%	4	18
26–50%	6	27
51–75%	3	14
76–100%	9	41
**Frequency of working with adults diagnosed with brain tumour**		
Frequently—at least once per month	5	23
Very frequently—at least once a week	5	23
Almost always—at least once per day	12	55
**Phase of illness support provided**^c^		
Pre-diagnosis	2	9
Diagnosis	14	64
Treatment/treatment coordination	19	86
Post treatment/ancillary	16	73
Primary physician	5	23
Palliative phase	13	59
**Frequency patient/carer report personality or behaviour change**		
Rarely (~ 10–30% of patients seen)	1	5
Sometimes (~ 30–60% of patients seen)	6	27
Most of the time (~ 60–90% of patients seen)	14	64
Always (~ 100% of patients seen)	1	5
**Frequency clinician screen/assess for personality or behaviour change**		
Never (0% of patients seen)	1	5
Rarely (~ 10–30% of patients seen)	2	9
Sometimes (~ 30–60% of patients seen)	5	23
Most of the time (~ 60–90% of patients seen)	6	27
Always (~ 100% of patients seen)	8	36

^a^Cancer care coordinator role comprises mainly nurses

^b^Full time equivalent

^c^Multiple responses possible

**Table 2:** Carer sample characteristics

	Australia (*N* = 7)	Denmark (*N* = 6)
**Carer characteristics**		
**Sex**		
Male	0	1
Female	7	5
**Age (median, range)**	61 (43–68)	55 (42–67)
**Education**		
High school	0	4
Postsecondary education	7	2
**Employment status**		
Employee or self employed	5	2
Retired/pension	1	1
Sick/carer’s leave	1	3
**Length of caregiving**		
0–6 months	1	0
7–12 months	4	2
> 12 months	2	4
**Bereaved carer time since patient death**		
0–6 months	1	1
7–12 months	1	0
> 12 months	2	0
**Relationship to patient**		
Spouse/partner	7	5
Sibling	0	1
**Marital status**		
Married/partner	3	6
Widowed	4	0
**Place of birth**		
Australia	5	0
Europe	1	6
North America	1	0
Children living at home*	3	4
**Patient diagnosis information**		
**Age (mean, range)**	55 (45–65)	53 (44–66)
**Tumour histology**		
Glioblastoma	6	6
Astrocytoma	0	1
Oligodendroglioma	1	0
**Tumour location**		
Frontal	3	2
Other	4	4
**Tumour site**		
Left side	5	3
Right side	1	1
Bilateral	0	2
Missing	1	0
**Tumour grade**		
III	5	1
IV	2	6
**Neurosurgical intervention**		
Total or partial resection	5	5
Biopsy	2	1
Treatment stage**		
Radiation therapy	1	0
Chemotherapy	1	0
Combined chemoradiation	5	6

*Yes/no response

**More than one can apply

### Procedures

Participants were recruited purposively using snowball recruitment methods. Potentially eligible HCPs and carers were recruited by advertising the study via national Australian brain tumour organisations (Cooperative Trials Group for Neuro-Oncology, The Brain Cancer Group, Brain Tumour Alliance Australia, Psycho-oncology Cooperative Research Group, and Peace of Mind Foundation) and by the healthcare team in Denmark from the neuro-oncology outpatient clinic. All participants provided written and verbal consent.

### Data collection

Patients’ clinical and personal characteristics were obtained by carer report in Australia and from the patients’ health records in Denmark. Carer socio-demographic and HCPs’ clinical experience data were collected via survey in Australia. A semi-structured interview guide was used to explore how carers and healthcare professional participants described BTrPBc and their impact on carers. Interviews were audio recorded and transcribed verbatim using transcription software Trint [9] and Viceron [10].

The research team undertook a thorough process to check, amend, and de-identify the transcripts. For the Danish interviews, interviews were translated into English using Viceron’s translation software [10]. Subsequently, members of the research team (SN, SK, KP) reviewed the transcripts while simultaneously listening to the original audio recordings. This approach enabled the team to detect and correct inconsistencies between spoken dialogue and AI-translated text, enhancing the accuracy of the transcripts.

### Analysis

Definition data were analysed deductively in relation to the Personality Change Due to Another Medical Condition diagnostic category [2] and inductively to uncover new ideas related to descriptions of BTrPBc as perceived by carers and HCPs. Inductive thematic analysis was conducted to develop themes exploring the impact of patient personality and behaviour changes on carers.

Data were analysed separately for HCPs and carers, then pooled for analysis based on the research questions. Thematic analysis procedures were used [11]. EM familiarised herself with the data by listening to the audio recordings of interviews and checked this against the transcription for accuracy. Subsequent review of transcripts involved generating initial codes and developing these into themes. Through the process of team discussion and review of the data, themes and sub-themes were refined to produce the final results. The Consolidated Criteria for Reporting Qualitative Studies (COREQ) checklist was used to guide reporting [12].

## Results

We interviewed 22 HCPs and 13 carers. Individual interviews were conducted via video-call (17 HCPs; one carer), telephone (four HCPs; eight carers), or in-person (one HCP; four carers). See Table 1 for HCP characteristics and Table 2 for carer demographics.

### Aim 1: Definitions and descriptions of brain tumour–related personality and behaviour change

Two themes were developed from carer and HCP descriptions of patient BTrPBc: (1) variable presentation and multifactorial aetiology and (2) manageable vs. challenging changes. HCPs and carers described BTrPBc as highly individual, with no singular pattern or cause and with changes presenting differently for each patient. Some changes were reported as subtle, others extreme, and with the potential to fluctuate over time. There were many identified contributing causes, such as the location of the tumour, medication side effects, cognitive impairment, emotional distress, fatigue, or sleep problems, with no single factor fully explaining the changes. Together, this reflects how complex, unpredictable, and unique these changes are across individuals and the need for nuanced understanding and flexible support tailored to each patient and their context. The results are presented in Table 3 with illustrative quotes.

**Table 3:** Definitions and descriptions of brain tumour–related personality and behaviour change

Theme	Theme description	Illustrative quotes
**Variable presentation and multifactorial aetiology**	HCPs commented on varying terms used to describe BTrPBc	*“Whether you want to call it behaviour or you want to call it cognitive or neurocognitive implications of disease, it just depends on the language you’re* *using and what discipline you come from.”* HCP021, Cancer Care Coordinator
	Fatigue and cognition were recognised as contributing to personality changes	*“He also wasn’t sleeping well, so I think prior to his tumour being removed and prior to us going away, his sleep was pretty poor, and he was only getting a couple of hours each night.”* C007, Aus
		*“He's always frustrated easily because he can’t understand people.”* C011, Aus
		*“I think it's fairly universal the majority of patients would have some degree of behaviour change… it is quite linked to cognitive dysfunction.”* HCP005, Radiation Oncology Nurse
	Carers identified different types of personality changes and that these changes were not static.	*“I think there were four waves of change… pre-diagnosis hyper-vigilance… then really negative… now she’s actually very happy, and that’s mainly due to the fact that she’s lost her short-term memory… she’s in this total space of gratitude.”* C003, DK
	Some HCPs and carers had observed extreme changes in patients	*“He was a very placid, softly spoken individual…He suddenly developed this really aggressive frontal brain cancer and the wife said to me ‘I don’t know who he is. This is not the person I married. This is, going from one extreme to the other and I don’t know how to deal with that emotionally’, because it was so out of normal for him.'”* HCP017, CCC
		*“He was very competent… one of the things that I fell in love with was how funny he was. He definitely changed… he became very frustrated and impatient.”* C011, Aus
	HCPs identified that sometimes patients notice changes in their own behaviours and thoughts	*“Sometimes it comes from the patient that will say to you, ‘I’ve noticed, I’m not the same’… or ‘I feel like I’m in a haze’.”* HCP013, CCC
**Manageable vs. challenging changes**	BTrPBc were discussed by HCPs and carers as ranging from manageable to challenging, depending on the clinical presentation of the BTrPBc and the effect of the change relationally	*“I guess the other way I would define it is if it is causing distress in the family with a carer or distress to themselves.”* HCP001, CCC
	Carers struggle with sustained/unexpected symptoms	*“Sometimes I have the ability to take that [personality change symptom of patient perseveration of speech] on and sometimes I don’t. Lately, I haven’t been able to.”* C011, Aus
	Carers spoke of the unexpected nature of personality changes and emergence of psychiatric presentations (mania, psychosis, etc.), which added to their sense of being overwhelmed	*“No one told me that this could be a potential [outcome] because you’re thinking cancer. You’re not thinking of mental health in the way of like manic, psychosis or anything.”* C007, Aus
	HCPs shared that not all personality and behaviour changes require intervention and emphasised the importance of assessment to determine the extent of support required	*“What’s a behaviour of concern for one person might not necessarily be for another… it’s about asking a few questions to elicit is it a behaviour of concern really.”* HCP001, CCC
	Support may be directed towards helping carers understand behaviour change	A HCP recalled carers asking:*" ‘Is it normal? because I've noticed a certain kind of behaviour,’ ‘Is this just temporary or will it get better?’”* HCP013, CCC
	HCPs spoke of providing support to help carers understand particular symptoms such as reduced motivation or apathy, and supporting them to adjust expectations and develop understanding	*“Personality or behaviour changes aren’t always a problem… it’s a problem for everybody else, but it's not a problem for that person. So why would we be trying to fix it if it’s not a problem for that person?… if the person themselves is okay with it, then perhaps it’s just supporting people to understand that.”* HCP021, CCC

### Features and symptoms of personality and behaviour change

Table 4 provides a summary of HCPs’ and carers’ definitions and descriptions when asked to define BTrPBc and the types of changes they have observed in patients with a brain tumour. The described personality and behaviour changes are categorised under DSM-5-TR specifiers and contrasts the clinical viewpoint (HCPs) with caregiving experiences of carers.

**Table 4:** Types of personality and behaviour changes mapped to the DSM-5-TR specifiers

DSM-5-TR specifiers	Keywords used in interviews	Quotes from HCPs	Quotes from carers
Emotional lability	HCPs: moody, manic, erratic, intense	*“Sometimes it’s about that sort of high level of reactivity in situations so that people are a little emotionally ungoverned and will react, get very agitated or react very quickly.”* HCP001	*“He was just again manic talking to everybody on the train”* C007, Aus *“He pendulums a little, he swings a little…Then it can be just about everything”* C006, DK
	Carers: manic, mood swings		
Disinhibited	HCPs: impulse control, limited/lack of insight/awareness, filter not there/lost their filter, lack of social filter, sexually inappropriate, loss of empathy, blunt, inappropriate	*“It could be as simple as just expressing themselves a little bit more readily than they would have in the past. That a filter is no longer there and so they might have had some of these thoughts before, but now they’re acting them out. So, it’s more that disinhibition, that frontal filters no longer there, they will just say exactly what they think, do what they want, be any way they want to, without actually thinking.”* HCP003	*“My concern right now is that he repeatedly, and he has done that regularly, that when he gets so mad, he wants to split [divorce].”* C005, DK *“She’s got this sort of hyper-speed-talking here [that] turned into an intense need to control her child. It’s been very painful to watch for the rest of us.”* C003, DK
	Carers: Impulsivity, controlling, child-like		
Aggressive	HCPs: frustrated, quick to anger, short tempered, aggressive, irritable, snap easily, less tolerant	*“I’ve seen quite a bit, people, a lot more short-tempered and get frustrated quite easily, which you know when people can’t do things they used to be able to do the frustration definitely builds up very quickly.”* HCP002	*“The verbal aggression and violence isn’t ever thought about in the carer realm from having none of this beforehand, to then having absolute rage, and I’ve never seen rage like zero to 100 rage, and it would happen.”* C007, Aus *“If we had guests over, and I didn’t interrupt my conversation at the minute [patient] wanted to say something, he got annoyed with me, because he’d forget what he wanted to say.”* BC002, DK *“He got a bit more aggressive initially and, like, he had a mean side to him came out.”* BC013, Aus
	Carers: arguing, aggressive, reacting, angry, frustrated, verbal abuse		
Apathetic	HCPs: anhedonia, adynamia, withdrawn, amotivation, depressed, apathy, [low] energy, fatigue, non-communicative, poor initiation	*“The other thing sometimes people find hard is sort of adynamia or apathy or, you know, poor initiation type behaviour can be really hard for families and misunderstood as laziness.”* HCP001 *“Change in interest as well, not finding things enjoyable anymore, maybe feeling withdrawn, maybe changes to the patterns when they’re socialising.”* HCP019 *“…* *I see a lot of depressed affect. I see a lot of apathy, energy, fatigue type issues*.” HCP020	*“He is sad, he misses everything about his old life. He has been depressed for some time.”* C004, DK
	Carers: depressed, negative		
Paranoid	HCPs: psychosis, paranoia, irrational	*“He and his wife had just split up because he threw her out because he said that she was after his money, she was trying to poison him slowly and different things – she wasn’t. I mean, we haven't found any reason to suggest this, and she seems to be quite good carer. So. he really doesn't have any insight into caring for himself.”* HCP007 *“So, it’s very difficult for a family member when someone has become psychotic or manic. And, you know, their general behaviour is actually, you know, highly unsafe for other family members because they’re misinterpreting things or they they’re actually just aggressive.”* HCP012	*“He was so afraid of how he was, in that moment, going to respond to a potential intruder. Like, that is not like him…he would never have that flight response to that sort of extent.”* C007, Aus *“Because he used to sometimes say to me, ‘you just want me to die’. I mean, that was really hard because that was the last thing I wanted to happen. Seriously.”* BC009, Aus
	Carers: paranoia, psychosis, suspicious		
Other	HCPs: cognitive/executive changes, poor intake of information, executive dysfunction, cognitive impairment	*“There’s a lot of cognitive impairment and that changes people a lot. It can be cognitive impairment because they have short memory issues or they’re forgetful and also depending on which part of the brain that is they had the resection. The family might just report that they are a different person, probably very teary, crying a lot, easily being irritable. And then sometimes the family just finding it difficult to explain things for the patients to understand that.”* HCP013	*“If I’ve been out shopping, and I’ve just been gone for, I don’t know, 40 min, and if I just went to two places, he’s overreacting like that, because he says I’ve been gone all day, without calling.”* C004, DK *“At work, he was losing it a little bit, you know, lack of focus, making some mistakes, which was highly unlike [him].”* BC010, Aus *“The first lot of surgery he was on steroids and he was very, up and about and wanting to do stuff, but of course he didn’t have the energy to do it.”* BC008, Aus
	Carer: time blindness, fatigue		
	HCPs: suicidal ideation	*“I do see infrequently but very high-risk suicidal ideation and self-harm behaviour in people with primary brain tumours. Oftentimes, people who have had essentially an injury to their frontal lobe, they’ve got an acquired brain injury, they can be quite absolute in their planning. So, if they feel like, you know, it would be better to die than to feel like this, and they can be very pragmatic about that, they don’t necessarily at that point in time have an ability to access, ‘well, what might it do to my family if I were to suicide or harm myself?'”* HCP012	*“Just do not always want to live anymore. So that’s also something I listen to a lot. He says this is no life for him.”* C004, DK *“He reacts by saying ‘I just have to find a place where I can die’ and it’s not fun for us to listen to.”* C006, DK
	Carer: not live anymore, want to die		

### Aim 2: Impact of patient personality and behaviour changes on carers

Three themes with sub-themes were identified related to the impact of patient BTrPBc on carers, based on HCP and carer interviews.

### Theme: Grieving the person: “I don’t know who this person is anymore”

HCPs and carers described carers as experiencing a sense of powerlessness and grief from witnessing changes to patients’ functioning and “Insight. When they’re not really aware of the changes in themselves, that can be difficult.” (HCP009, neuropsychologist).

HCPs and carers acknowledged the grief carers experience: “[carers] say to me ‘I don’t know who this person is anymore.’” (HCP005, radiation oncology nurse).

“Grief. That’s what it’s like. You know, this is going to happen and you’re watching somebody decline in front of your eyes and there is nothing you can do.” BC009, Aus.

“I think it is when they lose someone who’s always been loving, attentive, and kind and that person becomes harsh and critical and sometimes verbally aggressive,they’ve lost the person.They're really going into that grieving process of the person’s still alive, but they're grieving the person they love.” HCP008, clinical psychologist.

#### Subtheme: Grieving the relationship: “There is no marriage anymore”

Spousal carers described oscillating between their identity as a spouse and carer, by stating “My marriage is gone. There is no marriage anymore” (C012, Aus) and:

“I think it’s sort of created this feeling of being with someone that I didn’t really know any longer. Our relationship, I feel, became strained in a way.” BC013, Aus.

HCPs recognised the increased burden related to caring for someone who is different from who they married:

“I think that, for carers, often the hardest thing is that you sign on in sickness and in health. Generally speaking, most of my carers are willing to take that on, but it becomes a bit more of a challenge when they’re caring for someone who just is not the same person that they married.” HCP005, radiation oncology nurse.

Carers reflected on the feelings of loss or imbalance from relational shifts and changing roles from the impact of brain tumour diagnosis:

“There is also something about, that you want to be equal. But the situation does not really call for equality in this. There is no equality in this.” C005, DK.

“I guess I became a bit more almost like a mother to him, making sure that he took his medication and making sure that he ate antioxidants.” BC013, Aus.

Carers struggled to find a balance between allowing patient autonomy and intervening to prevent harm:

“The hard part was trying to enable his independence. [I was] walking behind him. But then he was so proud… and then you would think, hang on a minute, hands off. Let him just do what he wants to do. But you could see the train crash happening.” BC010, Aus.

Other carers experienced a strengthening of the relationship and closeness after diagnosis: “He [patient] isgood at comforting me, he tells me how much he loves me and how proud he is of me and if I wasn’t there and went through this with him, he wouldn’t be here.” C006, DK.

### Theme: Bearing the brunt of behavioural changes: “Happening behind closed doors”

Carers described isolation related to the covert nature of BTrPBc as something that occurs at home that may not be visible to others:

“It’s a different face when he goes out in public. People don’t understand how he really is. ‘Oh, he is doing great. Look at him’…But then he still has cognitive issues. So,I remind people of that.” C011, Aus.

“I just wish that there was some way that the oncologists could acknowledge these behaviours that can happen and emotional impact on carers. Even though we’re not the patient, I’m still living with it.” C012, Aus.

Often, it is the carers who are recipients of patient behaviours:

“Regardless of what you’re doing, you can jump and dance, and then there’s nothing. Then you'll be the target for the anger, for the frustration, for his sadness.” C005, DK.

“He’s a lot more outgoing with strangers and putting on a really goodfaçadeand he is not very nice behindcloseddoors.” C012, Aus.

HCPs also recognised carers may be recipients of patient behaviours:

“I think that’s really distressing for carers and often any sort of aggressive or agitated behaviour tends to be seen more by the carers because the patients are more comfortable with them so they can be more themselves or they’re with them more as they see this sort of trajectory of behaviour rather than just being on their best behaviour when they’re seeing the care coordinator or the oncologist.” HCP001, CCC.

These behaviours “behind closed doors” may exacerbate carers’ isolation yet go undetected by HCPs:

“Perhaps the carer is not volunteering that it's happening, that there are these personality and behaviour changes happening behind closed doors.” HCP006, CCC.

A carer provided insight that it may not be for lack of wanting to disclose the BTrPBc and ask for support: “[Patient] will not let me ask questions at the oncology appointment. So,that’s another behavioural change. He will not allow me to ask questions about treatment or anything.” C012, Aus.

Another carer perceived it as burdensome to seek support:

“I don’t want to call someone and disturb them. That’s how I work, I don’t want to call the nurse to ask her things… but I did anyway, I had to do it.” BC013, Aus.

Aggression and violence “behind closed doors” can be difficult for carers to disclose to HCPs:

“Domestic violence becoming an issue…and then because it’s not been him and you can't really share it as well.” C007, Aus.

#### Sub-theme: Aloneness: “It can make you feel lonely”

Carers described experiencing loneliness in their role, contributed by the emotional impact of disconnection due to their partner’s behaviour changes: “It can make you feel lonely if you don't get to experience your partner understand your thoughts and feelings.” C001, DK.

This carer recognised that the intensity of their experience may overwhelm others, leading them to withhold talking to others for support. This selective sharing creates a cycle of loneliness and isolation:

“It is isolating because you can’t tell everyone everything that's going on. It’s just too much for everyone.” C012, Aus.

Other carers felt their aloneness was caused by others’ discomfort or reluctance to ask them personal questions, possibly out of fear, uncertainty, or shyness:

“Not everyone is interested. Not everyone asks you about the deepest things. It’s probably because they are too shy…It’s often the case that people are in doubt about how to handle a disease like this and they are afraid to ask.” C001, DK.

HCPs described the impact of this workload, particularly in relation to the increased surveillance required when a patient has cognitive changes:

“Not being able to be left alone is often the other challenge of just knowing that this person inherently has poor judgement and is high risk because they might wander off.” HCP012, psychiatrist.

#### Theme: Safety concerns and aggression: “I didn’t know what was going to happen next”

The most impactful and difficult personality and behaviour changes were instances of aggression, disinhibition, or mania, which raise safety concerns: “I think the safety concerns are obviously paramount, if an individual is being physically aggressive.” HCP012, psychiatrist.

Participants acknowledged the causal role of the tumour, medication, and other treatments in behaviours uncharacteristic of the patient’s personality before the tumour. Both HCPs and carers described crises where patient behaviour had escalated to the point of requiring emergency services or hospitalisation:

“I've had incidents where like it’s almost been domestic violence where I’ve actually had to ask the carers to call the police because they feel unsafe with the husband (patient) and that was found to be a mixture of the brain cancer as well as the Keppra that he was on.” HCP017, CCC.

“So, it got into a massive, verbal tussle that he was just going to take the car and drive the kids. I thought he was going to…So I was having to call the police. Then the ambulance came, and the police came.” C007, Aus.

HCPs and carers emphasised the emotional and physical toll on carers, particularly female spouses, as they navigate fear, uncertainty, and conflicting feelings surrounding aggressive behaviours:

“I think the most difficult thing is aggression, agitation or anger. I think predominantly that behaviour really comes out in our male patients, so it’s the female carers that are dealing with that. On more than one occasion we’ve had to admit somebody with agitation because of those issues.” HCP014, CCC.

“The nurse said ‘Are you scared? Are you ever in fear?’ I was at one time. I remember him slamming down his fist on the bench top. I’ve never really seen him like that. It was scary because you just didn’t know. He was a very gentle man, and I didn’t know what was going to happen next.” BC009, Aus.

##### Sub-theme: Children and family safety: “I don't want to give [child] to him alone.”

When patients exhibit irritability, aggression, and mania, spousal carers described increased surveillance and protectiveness over their children:

“Our problem right now is that he wants [child] alone, and I don’t want to give [child] to him alone.” C005, DK.

“I was worried about him going on the train with the kids because he’s manic. I just don’t know where his cognitive thinking is going to be.” C007, Aus.

“But the other night, we had an episode where [young child] was sitting in her chair, and we were having dinner. Then she just starts testing the boundaries. She did everything she wasn’t supposed to do with dinner. I can really feel that he’s [patient] getting more and more triggered by it. In the end, he grabs her arm, and I can see that he’s squeezing it. I immediately say to him,‘You can stop that right now.’” C005, DK.

HCPs were also aware of the lasting impact patient behaviour can have on young children:

“The stuff I worry about, is if you’ve got a 5,6,7-year-old. I don’t want them to be distressed now and end up with trauma that carries on and in their adult life.” HCP006, CCC.

HCPs reported there were also difficulties in accessing social work support when violence was present:

“The family violence social workers were not used to the patient with the cancer being the perpetrator. It was just one of those things that was just really hard.” HCP001, CCC.

## Discussion

The findings highlight the profound grief, loneliness, and in some cases, safety concerns experienced by carers of patients with BTrPBc. Zwinkels et al.’s [3] definition of behavioural and personality changes in adults with glioma provides a useful framework. Based on our findings, however, we suggest expanding the definition to encompass the psychological and social factors contributing to personality and behaviour changes in people with a brain tumour. Participants recognised a range of contributing factors such as the adjustment and psychological impact of a brain tumour diagnosis, prognosis, and functional impairments; patient’s pre-existing coping mechanisms; relational dynamics; and socio-environmental stressors. These factors interact with the neurological impact of the tumour, influencing the severity and presentation of personality and behaviour changes.

A theme identified in this study was the grief carers experienced when the patient has personality changes. Participants described grieving not only the anticipated death, but also the loss of the person their loved one used to be. The concept of ambiguous loss has been widely discussed in the caregiving literature, particularly in the context of neurodegenerative diseases such as dementia [13] and amongst cancer carers [14], and seems to apply to carers of brain cancer patients. The profound and multiple losses associated with brain tumour are acknowledged as driving substantial existential distress [15, 16].

Carers in this study reported that the psychosocial impacts of personality changes were largely unacknowledged, leading to deep loneliness and overwhelming carer responsibilities. This aligns with literature that highlights brain cancer carers’ constant vigilance to prevent harm [17] and their greater workload compared to other cancer carers [18]. Carers have previously reported feeling unsupported, misunderstood, and isolated from family and friends [19]. Carers in our sample described their struggles with personality changes occurring “behind closed doors,” with minimal external recognition or support. These hidden behavioural symptoms and the emotional labour of managing them appear to contribute to carer distress.

Personality changes can also go unnoticed due to patients’ lack of insight. Simpson and colleagues [20] found moderate agreement between patient self-reports and clinician evaluations, indicating that many PwPMBT can reliably assess their own cognitive abilities. However, family member proxy ratings aligned more closely with clinician assessments, highlighting the valuable perspective carers can offer [20]. This reinforces the need for better recognition and intervention to support carers with behaviours that can occur at home. Despite this need, current health systems make it challenging for HCPs to have discussions with carers without patients present. Early documentation of patient consent to discuss their health with a carer may facilitate these conversations.

We discovered spousal carers had major concerns regarding patients’ brain tumour related aggression toward their children. Carers shared that fears of unpredictable behaviour and the distress of navigating these changes led to protective actions, such as restricting a child’s time with the patient-parent. In some instances, patient behaviour escalated to a crisis requiring emergency services involvement. There is growing acknowledgement of the need for more research and clinical management of brain tumour–related aggression. Rosenlund [21] conducted a narrative review of brain tumours and cognitive disorders highlighting general safeguarding principles and legislation that can guide clinical practice. Risk assessment and safeguarding [22] should be integrated into clinical care and involve multidisciplinary team management to protect both patients and families. However, further research and clearer guidelines are needed to improve risk management.

Although the themes identified in this study were broadly consistent across participants from Australia and Denmark, social and healthcare contexts may have influenced experiences and perspectives. Both countries have government funded healthcare systems, providing universal access to cancer treatment and supportive care. Barriers to neuro-oncology supportive care and rehabilitation exist internationally [23] and support services can be fragmented [8]. Elsewhere, contextual differences may influence how carers navigate the healthcare system, access supports, and engage with healthcare professionals. Our sample size precluded a comparative analysis of the definition and impact of BTrPBc. Importantly, however, participants in both settings reported similar unmet needs regarding recognition of carer roles and opportunities for private discussion with HCPs, and there is an absence of clear guidelines for managing patient aggression internationally.

### Limitations

Several limitations should be acknowledged. First, no formal measures of patient BTrPBc were administered; the inclusion criteria were based on carer appraised personality changes in the patient. Second, while a patient contributed to consumer review of the results of this research, patients were not included as interview participants, which may have limited the diversity of perspectives. Finally, some carers were supporting individuals living beyond the median survival time for high-grade glioma and time since bereavement varied from seven weeks up to 10 years. This variation may have influenced carers’ recall and their perceptions of the changes associated with brain tumours.

### Clinical implications

Key recommendations and clinical implications are summarised in Table 5 and Box 1 shows the key findings.

**Table 5:** Clinical implications and key recommendations

1. Not all individuals with BTrPBc will require intervention for these changes. However, screening and assessment of the presenting symptom (type; onset; frequency; severity; impact on patient, carer, and family; context of occurrence, e.g., antecedent, behaviour, consequence; and contributing factors) are necessary before determining whether intervention is required
2. Carers can provide valuable collateral information about patient functioning to HCPs to aid in assessment and management
3. Carers would benefit from opportunities to have time alone to ask questions and share information with HCPs. While this may create some challenges for HCPs in managing patient confidentiality, the inclusion of carers ultimately enhances patient care. This issue may be mitigated by informing the patient at the beginning of treatment that involving support persons is common practice and provides benefits such as identifying unnoticed changes, assisting with medication management, and offering adequate support. Documenting patient consent in an early consultation to discuss their health with carers without the patient present may facilitate this
4. Carers often experience unacknowledged caregiving responsibilities and a sense of invisibility. HCPs should validate their role, recognise the potential for role strain, normalise help-seeking behaviours, and provide referrals for additional support when needed
5. Future research and training opportunities for HCPs to support carers with patient aggression and to develop individualized safety plans (e.g., identification of triggers and early warning signs for potential violence/aggression/escalation, de-escalation strategies, exit plans, contact details for relevant support services, including emergency services). HCPs should provide domestic violence support services and helpline information where indicated

**Table:** Box 1 Summary of key findings

• **Definition of BTrBPc:** The definition should be expanded to include psychological and social factors contributing to personality and behaviour changes in people with a brain tumour.
• **Impact of BTrBPc:** BTrBPc have a profound impact on carers’ wellbeing.
• **Future research:** There is a need for further research on screening tools, management strategies and clinical guidelines for BTrPBc.

## Conclusion

This study highlights the nuanced and complex nature of BTrPBc, underscoring the need to expand diagnostic criteria and integrate carers into the care team. Carers should have opportunities to discuss safety concerns without the patient present, particularly when there are concerns regarding safety. Future research should explore longitudinal designs to track these symptoms over time and test interventions tailored to this population. Several validated tools are available to screen for neuropsychiatric and behavioural symptoms following brain injury [24–26]; however, these measures have not been validated in brain tumour populations. Further research is needed for optimal screening for personality and behaviour changes in this cohort. Notably, patient aggression was a significant concern raised by HCPs and carers, yet there are no clear guidelines or processes for managing violence and aggression in this context. Research must prioritise testing interventions and support strategies to safeguard patients and their families.

## Data Availability

Data will be made available upon reasonable request.
